# Efficacy of Oxygen‐Enriched Platelet‐Rich Plasma Combined With Minoxidil in the Treatment of Androgenetic Alopecia: A Retrospective Study

**DOI:** 10.1111/jocd.71078

**Published:** 2026-07-16

**Authors:** Jianyun Lu, Tong Zhang, Dan Wang, Lu Zhou, Jian Huang, Zhen Tang, Guosheng Zhao, Yunfeng Fu, Hong Zhu, Lihua Gao

**Affiliations:** ^1^ Departments of Dermatology The Third Xiangya Hospital, Central South University Changsha China; ^2^ Department of Dermatology Xiangya Hospital, Central South University Changsha China; ^3^ Xiangya School of Medicine Central South University Changsha China; ^4^ Department of Blood Transfusion The Third Xiangya Hospital, Central South University Changsha China; ^5^ Department of Traditional Chinese Medicine The Third Xiangya Hospital, Central South University Changsha China

**Keywords:** androgenetic alopecia, hair regrowth, minoxidil, ozone therapy, platelet‐rich plasma (PRP)

## Abstract

**Background:**

Androgenetic alopecia (AGA) is a common disorder that negatively affects quality of life. Platelet‐rich plasma (PRP) has shown efficacy in hair regeneration. Ozone treatment amplifies the biological effects of PRP, thereby strengthening its role in follicular regeneration.

**Objective:**

To evaluate the efficacy and safety of oxygen‐enriched PRP combined with 5% minoxidil compared with minoxidil monotherapy in AGA.

**Methods:**

In this retrospective comparative study, treatment of 72 patients was reviewed. Patients were categorized according to the treatment they had received: oxygen‐enriched PRP combined with minoxidil or minoxidil monotherapy. The combination group received 5 PRP injection sessions at two‐week intervals with daily topical minoxidil, while the control group used minoxidil alone. Efficacy was assessed by dermoscopic parameters and satisfaction scores; adverse events were recorded.

**Results:**

The combination group showed significantly greater improvements in hair density, hair diameter, and follicular density compared with monotherapy (*p* < 0.05). No significant differences were observed in terminal hair ratio or mean number of hairs per follicular unit (*p* > 0.05). Patient and physician reported satisfaction scores were higher in the combination group. Adverse events were mild and transient, with no serious complications.

**Conclusion:**

Oxygen‐enriched PRP combined with minoxidil is more effective than minoxidil alone for improving hair growth parameters and patient satisfaction. These findings support ozone‐treated PRP as a promising therapy for AGA.

## 1 Introduction

Androgenetic alopecia (AGA) is a common form of hair loss, a chronic, progressive disorder. Although it does not affect physical health, it can significantly impair patients' psychological well‐being and quality of life [1]. It is characterized by follicular miniaturization, a shortened anagen phase, and a prolonged telogen phase. This leads to the gradual transformation of terminal hairs into vellus hairs, subsequently causing hair thinning and loss [2]. Currently, the US Food and Drug Administration (FDA) has only approved minoxidil and finasteride as limited pharmacological treatments for AGA. With increasing public attention on AGA, more innovative therapies are being proposed to improve patient compliance and treatment satisfaction.

In recent years, platelet‐rich plasma (PRP) has been widely used to promote hair follicle regeneration due to its abundance of growth factors (GFs). Studies have reported that PRP monotherapy can significantly improve hair density and diameter [3]. Concurrently, research combining PRP with 5% minoxidil has demonstrated efficacy superior to using either minoxidil or PRP alone [4].

Ozone (O_3_) is a gaseous molecule with strong oxidizing and bactericidal properties, offering potential therapeutic advantages in the medical field [5]. When properly processed and administered in appropriate concentrations, ozone can be used for various conditions, forming the basis of ozone therapy, which is widely applied in diabetic complications, lumbar disc herniation, oral diseases, dermatological conditions, and neurological disorders [6, 7]. When ozone is dissolved in PRP, it generates reactive oxygen species (ROS) that stimulate the release of multiple GFs, thereby enhancing tissue regeneration and promoting repair [8].

To date, no high‐quality clinical studies have systematically evaluated the efficacy of ozone‐treated, oxygen‐enriched PRP in treating AGA. Therefore, this study adopts a retrospective comparative design to evaluate the therapeutic effects of oxygen‐enriched PRP combined with 5% minoxidil versus 5% minoxidil monotherapy, aiming to provide real‐world evidence to support treatment strategies for AGA.

## 2 Materials and Methods

### 2.1 Study Design

This study is a retrospective comparative clinical study. Approved by the Ethics Committee (The third Xiangya Hospital of Central South University, Fast 22 160) and conducted in accordance with the Declaration of Helsinki. All patients provided written informed consent. Medical treatment of patients diagnosed with AGA and treated between February 2021 and May 2025 was reviewed. Eligible patients were categorized into two groups according to the treatment: oxygen‐enriched PRP combined with Minoxidil group (Minoxidil+PRP group) and minoxidil monotherapy group (Minoxidil group). Each group included 36 patients. The injection of PRP treatments was administered by the same surgeon, while periodic evaluations were performed by the same physician, who was unaware of treatment allocation during assessment.

### 2.2 Preparation of PRP

PRP was prepared using sodium citrate as the anticoagulant and an automated blood collection system (COM.TEC, Fresenius Kabi, Friedberg, Germany), according to the standardized cell separation protocol of the Department of Transfusion Medicine at the hospital. For each patient, 280 mL of whole blood was collected under sterile conditions. The system automatically analyzed whole blood components at 25°C and adjusted centrifugation parameters automatically to reduce red blood cell and leukocyte contamination while achieving a target platelet concentration approximately fourfold higher than baseline.

Under this standardized protocol, approximately 50–60 mL of PRP was obtained from each collection. The PRP was divided into five equal aliquots, with 10–12 mL allocated for each treatment session. One aliquot was used immediately for the first treatment session, whereas the remaining aliquots were stored at−80°C in the Department of Transfusion Medicine for subsequent treatment sessions. Its storage and thawing procedures are always carried out in strict accordance with the relevant management regulations for blood products.

The composition of PRP was characterized in a subset of patients by measuring platelet, leukocyte, and red blood cell counts before and after PRP preparation. These measurements were used to confirm platelet enrichment and assess the extent of leukocyte and red blood cell removal. The PRP composition data are summarized in Table 1.

**TABLE 1:** Hematological characteristics of PRP samples.

	WBC (×10^9^/L)	RBC (×10^12^/L)	HGB (g/L)	HCT (%)	PLT (×10^9^/L)
Participant 1	0.01	0.03	1	0.2	1001
Participant 2	0.09	0.01	1	0.1	1057
Participant 3	0.15	0.01	1	0.0	1032
Participant 4	0.00	0.00	0	0.1	955
Participant 5	0.01	0.00	0	0.0	1077
Participant 6	0.03	0.00	1	0.0	876
Participant 7	0.02	0.00	0	0.0	985
Participant 8	0.03	0.00	1	0.0	1061

Abbreviations: HCT, hematokrit; HGB, hemoglobin; PLT, platelet count; RBC, red blood cell; WBC, white blood cell count.

Preparation of oxygen‐enriched PRP was performed using ozone generated by a HUMAZON PROMEDIC ozone therapy unit (Humares GmbH, Bruchsal, Germany). Ozone concentration was adjustable with the device, and in this study, ozone gas at a concentration of 57 μg/mL was used. An equal volume of ozone gas was drawn into a syringe and mixed with an equal volume of PRP. The mixture was then placed on a shaker and gently agitated for 5 min to ensure sufficient mixing. Afterward, excess gas remaining in the syringe was expelled, and the ozonized PRP was immediately used for injection.

Female patients underwent PRP collection during non‐menstrual periods. All patients were evaluated for abnormal complete blood counts and coagulation function prior to PRP collection to exclude any hematological irregularities.

### 2.3 Inclusion and Exclusion Criteria

Patients with AGA, classified according to the Norwood‐Hamilton system, were retrospectively identified from the Department of Dermatology at The Third Xiangya Hospital of Central South University between February 2021 and May 2025.

#### 2.3.1 Inclusion Criteria

(1) Age 18–65 years; (2) Diagnosed with AGA at Norwood‐Hamilton stage II‐IV (males) or Ludwig stage I‐III (females); (3) Normal complete blood counts and coagulation function; (4) Provided informed consent and demonstrated ability to comply with the study protocol and follow‐up schedule.

#### 2.3.2 Exclusion Criteria

(1) Pregnancy or lactation; (2) Hematological disorders; (3) Use of anticoagulants within 1 year; (4) History of cutaneous malignancies; (5) Infections including HIV, syphilis, hepatitis; (6) Severe psychiatric or systemic organ diseases; (7) History of systemic or topical hair loss treatments within 1 year.

### 2.4 Treatment Protocol

#### 2.4.1 Minoxidil + PRP Group

Received injections once every 2 weeks for a total of 5 sessions. Injection sites targeted alopecic areas, primarily the M‐shaped regions in men and the vertex in women. For female patients with frontal hairline recession, injections were also administered in the M‐shaped regions. Prior to each injection, topical lidocaine cream was applied for 40 to 60 min. Then injecting a total volume of 8–12 mL of oxygen‐enriched PRP, starting with a mixture of 3.2 mL oxygen‐enriched PRP and 0.8 mL lidocaine. And then the injection was performed at a 1 cm interval at the injection area, followed by an intensive injection at the end, using 34G needles. All patients applied 5% minoxidil solution topically twice daily (1 mL/application) and massaged until absorbed.

#### 2.4.2 Minoxidil Group

Mirrored the topical minoxidil regimen of the Minoxidil + PRP Group, but no injections.

### 2.5 Assessment of Treatment

Patients underwent macro photography and dermoscope assessment of hair growth before each treatment session. Using the dermoscope (Moleanalyzer‐Pro, FotoFinder Systems GmbH, Bad Birnbach, German), the following parameters were measured within a 0.922 cm^2^ area: hair density, hair diameter, terminal hair ratio, vellus hair ratio, follicle density, and mean number of hairs per follicular unit. Measurements were averaged across 3 fields of view. At the final follow‐up, both patients and physician independently evaluated treatment efficacy using a 4‐point Likert scale (0–3): No improvement (0), Slight improvement (1), Moderate improvement (2), Significant improvement (3).

**FIGURE 1:**
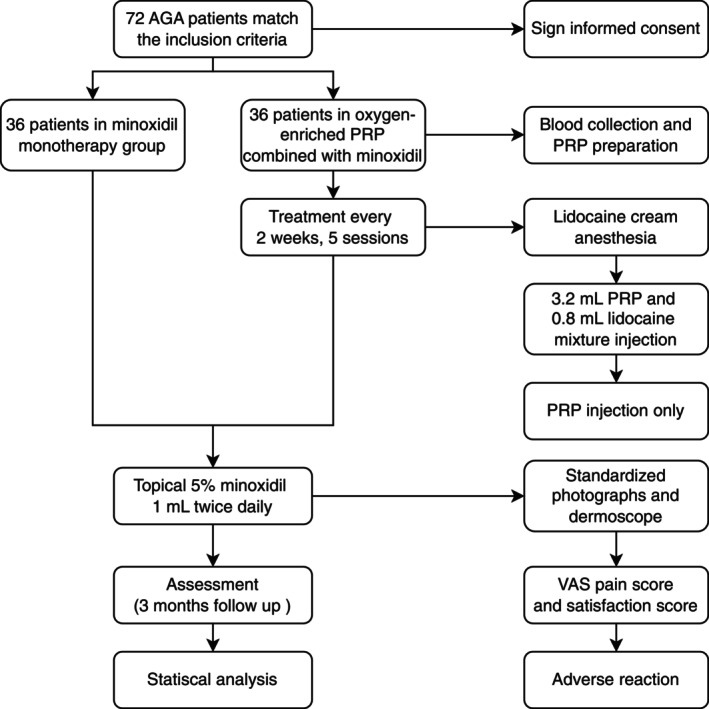
Study flow chart.

Post‐injection pain was assessed via Visual Analogue Scale (VAS), graded from 0 (no pain) to 10 (worst imaginable pain) on a numbered chart. Adverse events including local erythema, edema, and their incidence were recorded throughout treatment and follow‐up. After completing all 5 injections, patients were followed at 2 weeks, 1 month, and 3 months after the final injection. The overall study flow is presented in Figure 1.

### 2.6 Statistical Analysis

Statistical analyses were performed using GraphPad Prism 8, SPSS 23 and R software version 4.4.2. Continuous variables conforming to a normal distribution are presented as mean ± standard deviation (SD). Intra‐group comparisons were performed using the paired *t*‐test, while inter‐group comparisons utilized the independent samples *t*‐test. Data not conforming to a normal distribution were analyzed using the Mann–Whitney U test for inter‐group comparisons. Categorical variables are expressed as frequencies and percentages, and comparisons between groups were conducted using the χ^2^ test. Generalized linear models were constructed using the change rates of quantitative dermoscopic parameters as dependent variables. Change rate was calculated as the final follow‐up value divided by the baseline value for each participant. Age, sex, disease duration, and severity of AGA were included as covariates. *p* < 0.05 was considered statistically significant.

## 3 Results

Our study included 72 patients. Patients were categorized into two groups according to the treatment they had received in routine clinical practice: Minoxidil + PRP group (*N* = 36), receiving PRP injections combined with daily minoxidil therapy; Minoxidil group (Control, *N* = 36) receiving daily minoxidil monotherapy. The baseline characteristics of patients are summarized in Table 2. Patients in the Minoxidil + PRP group were significantly older than those in the Minoxidil group (35.6 ± 10.8 vs. 28.44 ± 6.25 years, *p* = 0.005) and had a longer disease duration (53.45 ± 21.32 vs. 33.84 ± 23.96 months, *p* < 0.001). Sex distribution was identical between groups, with 15 women and 21 men in each group. AGA classification was also comparable between the two groups. No significant differences were observed in baseline hematological parameters.

**TABLE 2:** Baseline clinical and hematological examination results of patients with androgenetic alopecia.

Characteristic	Minoxidil + PRP	Minoxidil	*p*
Age, Mean ± SD, years	35.58 ± 10.77	28.44 ± 6.25	0.005
Sex, *n*(%)			> 0.99
Women	15 (41.7%)	15 (41.7%)	
Men	21 (58.3%)	21 (58.3%)	
Duration of disease, Mean ± SD, months	53.45 ± 21.32	33.84 ± 23.96	< 0.001
AGA classification			
Male, *n*(%)			> 0.99
II	0	2 (9.5%)	
III	3 (14.3%)	4 (19.1%)	
III vertex	14 (66.7%)	12 (57.1%)	
IV	2 (9.5%)	3 (14.3%)	
V	2 (9.5%)	0	
Female, *n*(%)			> 0.99
I	13 (86.7%)	14 (93.3%)	
II	2 (13.3%)	1 (6.7%)	
III	0	0	
WBC (10^9^/L)	7.32 ± 1.43	7.88 ± 1.49	0.098
HGB (g/L)	140.00 ± 14.97	143.86 ± 10.61	0.265
PLT (10^9^/L)	251.43 ± 39.25	235.14 ± 36.70	0.128
RBC (10^12^/L)	4.75 ± 0.54	4.81 ± 0.27	0.608

Abbreviations: HGB, hemoglobin; PLT, platelet count; RBC, red blood cell; WBC, white blood cell count.

**FIGURE 2:**
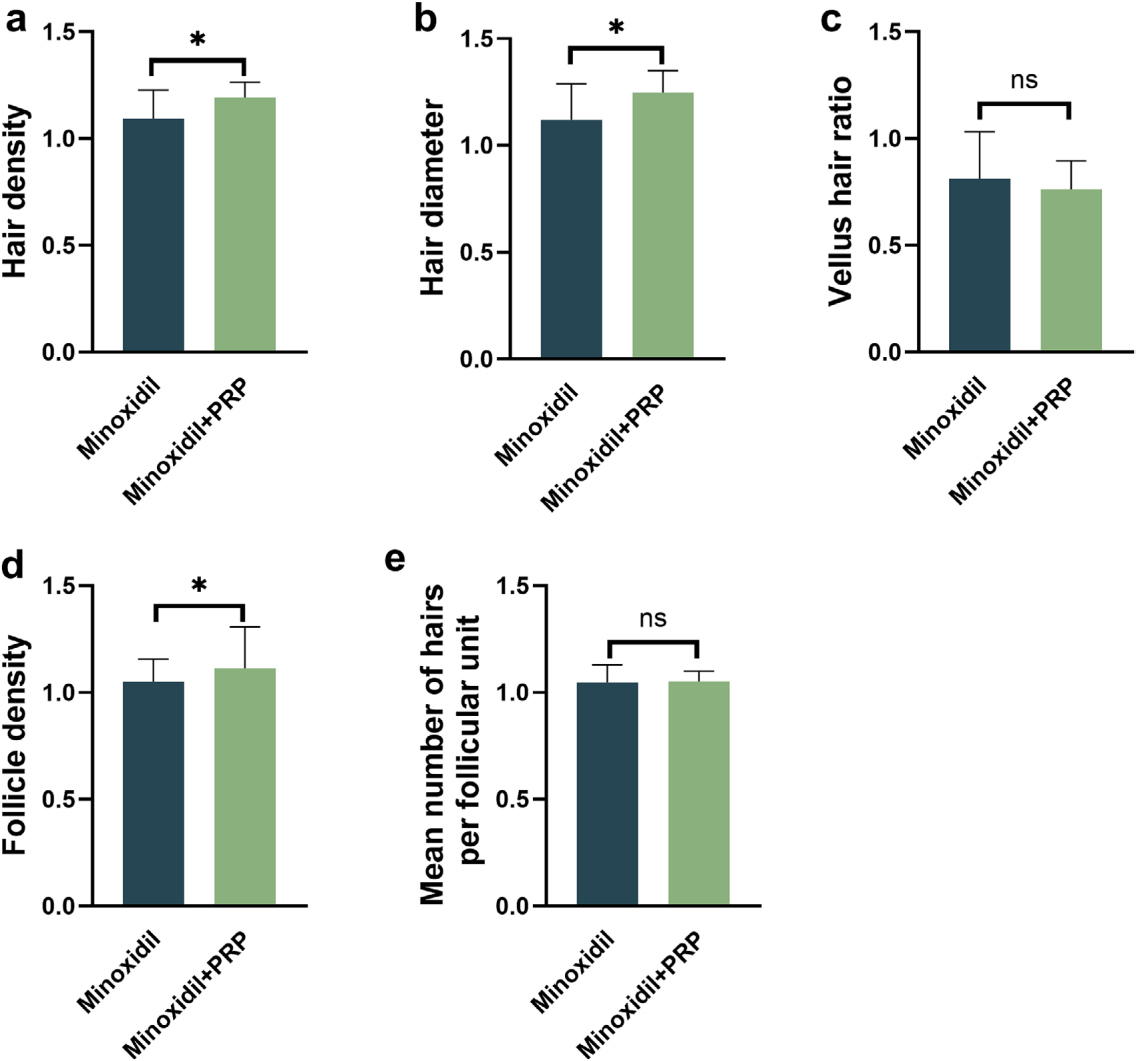
Comparative dermoscopic analysis of hair parameters before and after treatment. Minoxidil: Minoxidil monotherapy group. Minoxidil+PRP: Oxygen‐enriched PRP combined with minoxidil group.

**FIGURE 3:**
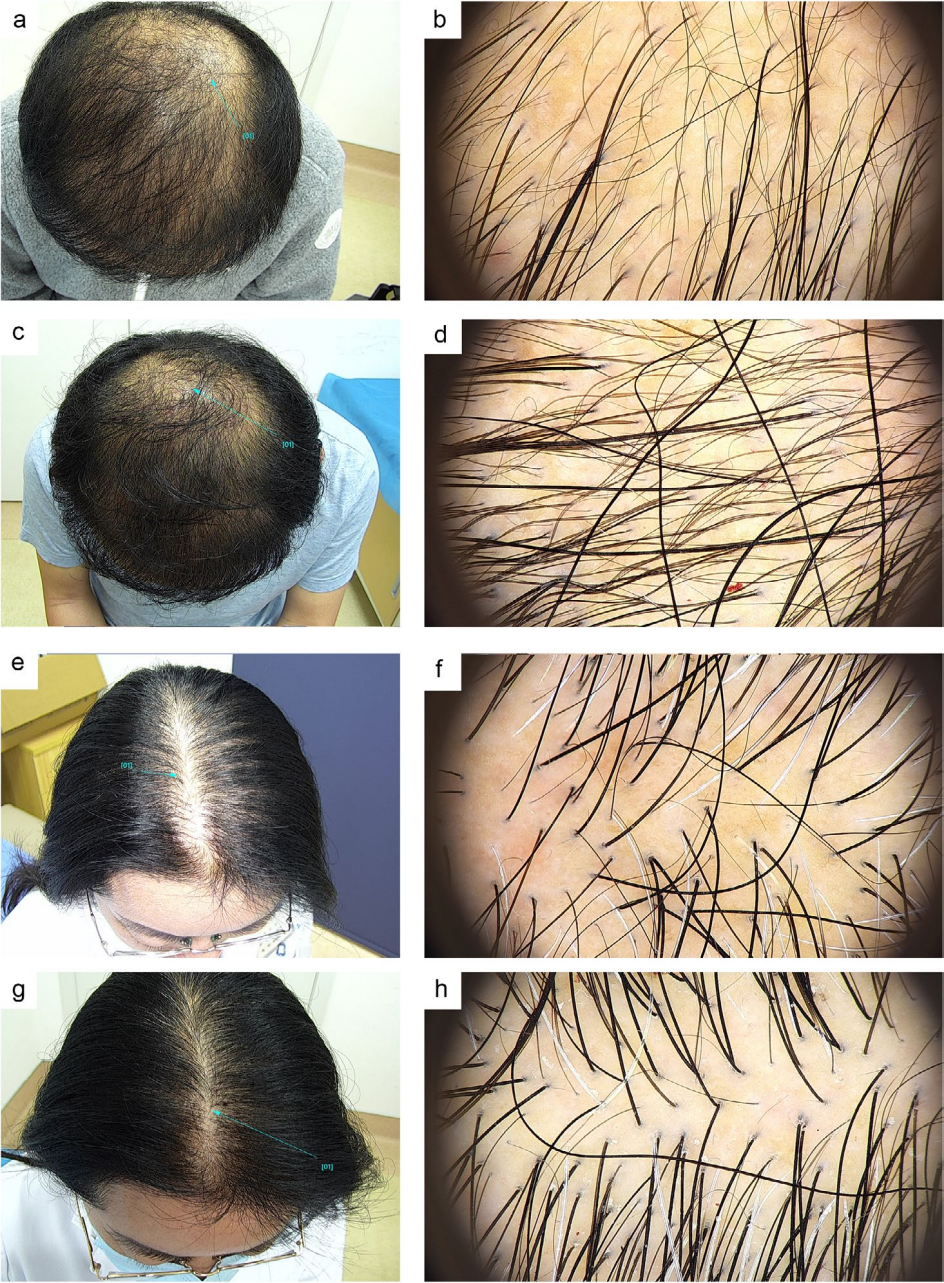
Macroscopic and dermoscopic images of 2 patients with AGA treated with oxygen‐enriched PRP combined with minoxidil. (A, a–d) A male patient: Baseline macroscopic (a) and dermoscopic (b) images, and three‐month post‐treatment macroscopic (c) and dermoscopic (d) images. (B, e–h) A female patient: Baseline macroscopic (e) and dermoscopic (f) images, and three‐month post‐treatment macroscopic (g) and dermoscopic (h) images.

**FIGURE 4:**
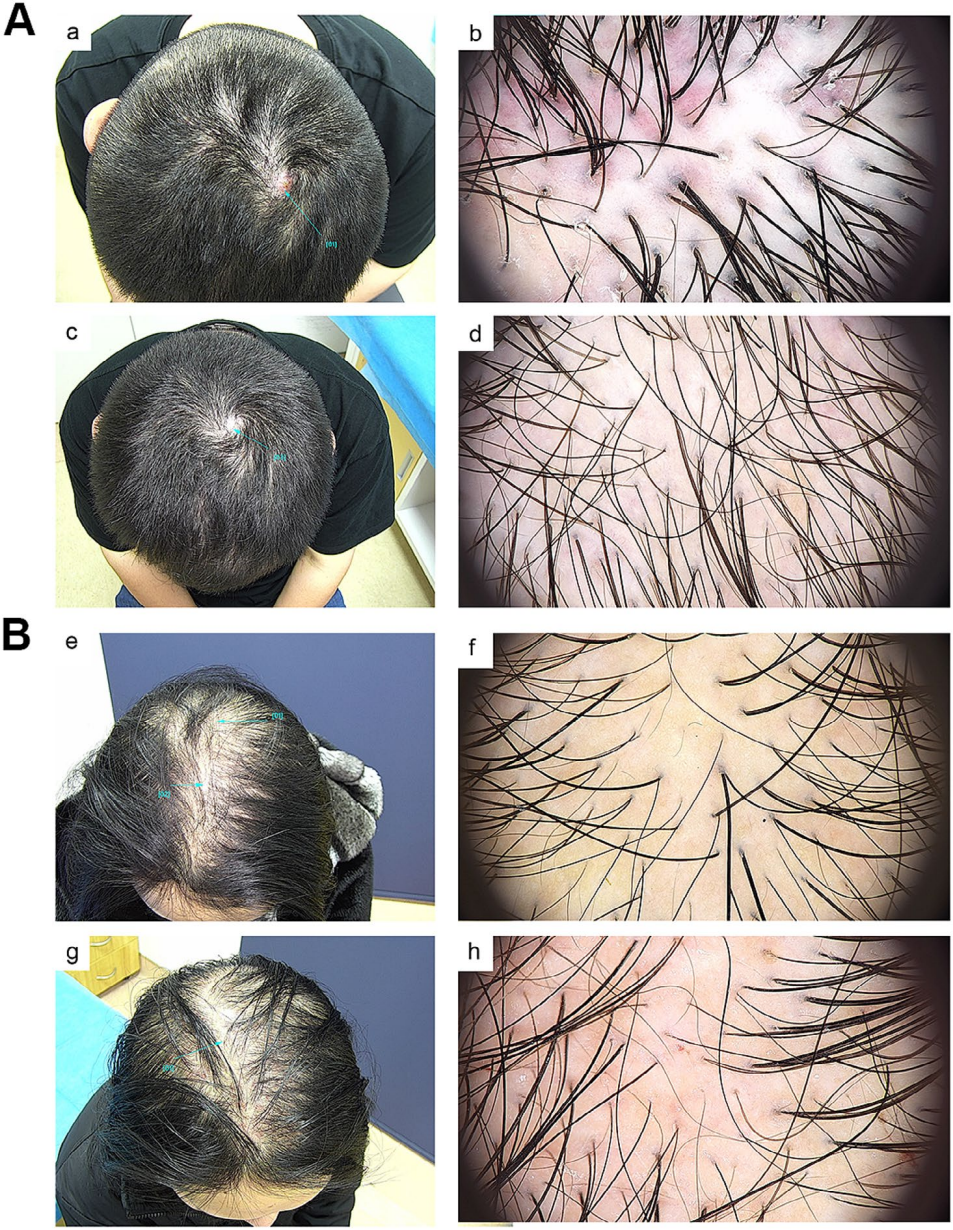
Macroscopic and dermoscopic images of 2 patients with AGA treated with minoxidil monotherapy. (A, a–d) A male patient: Baseline macroscopic (a) and dermoscopic (b) images, and three‐month post‐treatment macroscopic (c) and dermoscopic (d) images. (B, e–h) A female patient: Baseline macroscopic (e) and dermoscopic (f) images, and three‐month post‐treatment macroscopic (g) and dermoscopic (h) images.

The mean duration of alopecia in the Minoxidil + PRP group was 53.45 ± 21.32 months, significantly longer than the 33.84 ± 23.96 months in the Minoxidil group (*p* < 0.05). As shown in Figure 2 and Table S1, intergroup comparisons of dermoscopic parameters revealed statistically significant differences in hair density, hair diameter, and follicle density (*p* < 0.05). These three parameters showed greater improvement in the Minoxidil + PRP group compared to the Minoxidil group. However, no significant differences were observed in vellus hair ratio and mean number of hairs per follicular unit (*p* > 0.05). Macroscopic and dermoscopic photographs of patients in the Minoxidil + PRP group are shown in Figure 3. Corresponding images of patients in the Minoxidil group are provided in Figure 4.

To further adjust for potential confounding, generalized linear models were performed using the change rates of quantitative dermoscopic parameters as dependent variables, with adjustment for age, sex, disease duration and severity of AGA (Table S2). In these adjusted models, the Minoxidil + PRP group remained significantly associated with greater improvement in hair density compared with the Minoxidil group (β = 0.067, 95% CI: 0.012–0.123, *p* = 0.019). Similar significant associations were observed for hair diameter (β = 0.083, 95% CI: 0.011–0.156, *p* = 0.028) and follicular density (β = 0.147, 95% CI: 0.079–0.215, *p* < 0.001). However, the treatment group was not significantly associated with changes in vellus hair ratio or mean number of hairs per follicular unit. These findings indicate that the superiority of oxygen‐enriched PRP combined with minoxidil remained robust for hair density, hair diameter, and follicular density after adjustment for demographic factors and disease‐related factors.

According to patients' self‐assessed efficacy scores, in the Minoxidil + PRP group, 2 (5.6%) patients reported no improvement, 13 (36.1%) reported slight improvement, 17 (47.2%) reported moderate improvement, and 4 (11.1%) reported significant improvement, with a mean efficacy score of 2.56 ± 0.77. The mean physician assessed efficacy score was 2.53 ± 0.77, with no significant difference between patient and physician assessments (*p* > 0.05). Among patients receiving minoxidil alone, 12 (33.3%) reported no improvement, 18 (50.0%) reported slight improvement, 5 (13.9%) reported moderate improvement, and only one reported significant improvement; the mean efficacy score for these patients was 1.78 ± 0.68, and the mean physician score was 1.83 ± 0.61, with no significant difference between patient and physician assessments (*p* > 0.05). However, there were significant differences in both patient and physician scores between the two treatment groups (Figure S1).

Regarding adverse reactions, 3 patients in the PRP group experienced transient erythema, 2 had localized edema, and 1 developed scalp dermatitis after treatment, all adverse reactions resolved spontaneously; no serious adverse reactions occurred. The mean pain score for patients receiving PRP injections was 3.88 ± 1.19.

## 4 Discussion

AGA is a chronic and progressive hair disorder characterized by a prolonged treatment course and slow therapeutic response [9]. In clinical practice, continuous treatment for at least 6 months is usually required before an initial assessment of efficacy can be made. Meaningful improvement often requires sustained intervention for 12 months or longer. At present, monotherapies such as oral finasteride and topical minoxidil often provide only partial clinical improvement, and a substantial proportion of patients, particularly those with advanced AGA, fail to achieve satisfactory hair regrowth [10, 11]. Therefore, various adjunctive therapies are often combined with conventional pharmacological treatments to improve the overall response rate and shorten the time to visible improvement [12, 13]. This has led to the development of multimodal therapeutic strategies for AGA. Current adjunctive options include low‐level light/laser therapy (LLLT), microneedling, platelet concentrates such as platelet‐rich plasma and concentrated growth factor (CGF), exosome‐based therapies, and hair follicle mesenchymal stem cells (HF‐MSCs) [2].

LLLT is a non‐invasive physical treatment that may promote hair growth through photobiomodulation [14]. Its clinical effects are mainly reflected by increases in hair count or hair density [15, 16]. However, the improvement appears to be more evident in patients with mild‐to‐moderate AGA, and its efficacy may depend on device parameters, treatment frequency, and long‐term adherence [17]. Exosome therapy has also shown potential for improving hair growth [18, 19]. A multicenter study showed increased hair density after 12 months and demonstrated extracellular vesicles around the cellular component [20]. Hair follicle HF‐MSCs have also attracted attention because AGA scalps retain stem‐cell reservoirs but show reduced actively proliferating progenitors [21, 22]. Nevertheless, this clinical evidence remains relatively limited. Its long‐term safety is still uncertain, and regulatory standards have not yet been fully established.

Among these regenerative approaches, PRP is one of the most extensively studied interventions for AGA. In a randomized controlled study, Gentile et al. reported that three sessions of PRP treatment significantly increased the mean hair count and hair density in the target area [3]. Non‐activated PRP increased hair number and density [23], while activated PRP outcomes varied between preparation systems [24], underscoring the need to define the activation method and product characteristics when interpreting PRP efficacy [25].

In addition, PRP combined with standard medications or other adjunctive therapies has been widely used in clinical practice [12]. For example, randomized controlled studies comparing PRP plus minoxidil with minoxidil monotherapy in patients with AGA have shown that combined treatment can further increase hair density and diameter [26].

Against this background, the present study further introduced oxygen‐enriched PRP combined with 5% minoxidil for the treatment of AGA. Unlike non‐activated PRP or PRP activated by CaCl_2_ or thrombin [27], ozonized PRP may have a distinct biological profile. Ozone may induce platelet α‐granule degranulation and promote the release of multiple GFs, thereby enhancing the biological activity of PRP [28]. Besides, a transient oxidative stimulus generated by an appropriate dose of ozone may participate in antioxidant responses, inflammatory regulation, and local microcirculatory improvement [29, 30, 31].

In the present study, patients treated with oxygen‐enriched PRP plus minoxidil showed significantly greater improvements in hair density, hair diameter, and follicular density than those treated with minoxidil alone (*p* < 0.05). These findings further support the role of PRP in promoting follicular regeneration and improving hair quality. By contrast, no significant between‐group differences were observed in the vellus hair ratio or the mean number of hairs per follicular unit (*p* > 0.05). This suggests that the main benefit of the treatment may be related to improving the quality of existing follicular units.

It is worth noting that disease duration differed significantly between the two groups (*p* < 0.05). This mismatch may reflect the real‐world clinical decision‐making. Patients with a longer disease duration, stronger treatment demands, or insufficient responses to previous therapies are often more willing to receive PRP‐based combination therapy. Nevertheless, after adjustment for potential confounding factors, including age, sex, and disease duration, oxygen‐enriched PRP combined with minoxidil remained significantly associated with improvements in hair density, hair diameter, and follicular density. Based on the above mechanisms and our clinical findings, we propose that oxygen‐enriched PRP combined with minoxidil may be an intensified combination strategy. It may be particularly suitable for patients with insufficient response to conventional topical therapy, long disease duration, high treatment expectations, or a need for more evident improvement in hair quality.

The strengths of this study include its retrospective comparative design and the use of adjusted analyses to reduce the influence of baseline imbalances, including age, sex, disease duration, and AGA severity. In addition, the use of the COM.TEC automated blood component separation system in this study helped improve the standardization and reproducibility of PRP preparation. This approach also reduced the need for repeated blood collection, thereby improving clinical feasibility and patient compliance.

This study also has several limitations. First, because of its retrospective observational design, causal relationships cannot be established. Second, this study did not include comparator groups receiving other regenerative therapies. Therefore, it cannot directly determine the independent superiority of ozonized PRP over other PRP preparation methods or other adjunctive strategies. Future prospective randomized controlled studies with larger sample sizes and longer follow‐up periods are needed to further validate the efficacy and safety.

## 5 Conclusion

In conclusion, oxygen‐enriched PRP demonstrates promising potential as an effective therapeutic approach for enhancing hair regeneration. Compared with minoxidil monotherapy, the combined treatment of oxygen‐enriched PRP injections with minoxidil shows superior outcomes in improving both objective hair parameters and patients' satisfaction. Future studies should prioritize large long‐term follow‐up clinical trials to validate these findings, ultimately establishing a safer and more effective treatment protocol for AGA patients.

## Author Contributions

J.L. and T.Z. contributed to manuscript drafting. D.W., L.Z., J.H., and Z.T. participated in the research and data acquisition. J.L., H.Z., and L.G. contributed to study conception and design. Y.F. and G.Z. performed data curation and analysis. J.L. obtained funding. All authors participated in data collection, critically revised the manuscript for important intellectual content, and approved the final version for submission.

## Funding

This work was supported by the grants from the Natural Science Foundation of Hunan Province (2025JJ80143) and the Project of Health Committee of Hunan Province (20253862). These funds were used to support patient treatments and study‐related procedures.

## Disclosure

Artificial Intelligence Use Statement During the preparation of this manuscript, DeepSeek was used to assist with grammar correction and language polishing. It was used to improve clarity and readability. It was not used to formulate the research question, design the study, collect or analyze data, or interpret the findings and conclusions. All AI‐assisted text was carefully reviewed by the authors. The authors manually reviewed and finalized all AI‐polished scientific terminology and logic, and take responsibility for the accuracy, integrity, and originality of the final manuscript.

## Ethics Statement

The patients in this manuscript have given written informed consent to publication of their case details. Approved by the Ethics Committee (The third Xiangya Hospital of Central South University, Fast 22 160).

## Conflicts of Interest

The authors declare no conflicts of interest.

## Supporting information


**Figure S1:** Comparison of efficacy ratings between physicians and patients.
(A) Physician vs. patient ratings in the minoxidil+PRP group. (B) Physician vs. patient ratings in the minoxidil‐only group. (C) Intergroup comparison of patient ratings. (D) Intergroup comparison of physician ratings.

**Table S1:** Baseline, final follow‐up visit, and change rates of quantitative dermoscopic parameters in the Minoxidil + PRP group and the Minoxidil group.
**Table S2:** Adjusted generalized linear model analyses of change rates in quantitative dermoscopic parameters.

## Data Availability

The data that support the findings of this study are available from the corresponding author upon reasonable request.
