# Supplementary figures and images for: Efficacy of Oxygen‐Enriched Platelet‐Rich Plasma Combined With Minoxidil in the Treatment of Androgenetic Alopecia: A Retrospective Study
Source: J Cosmet Dermatol. 2026 Jul 16;25(7):e71078. doi: 10.1111/jocd.71078 (PMC13377007; doi:10.1111/jocd.71078)

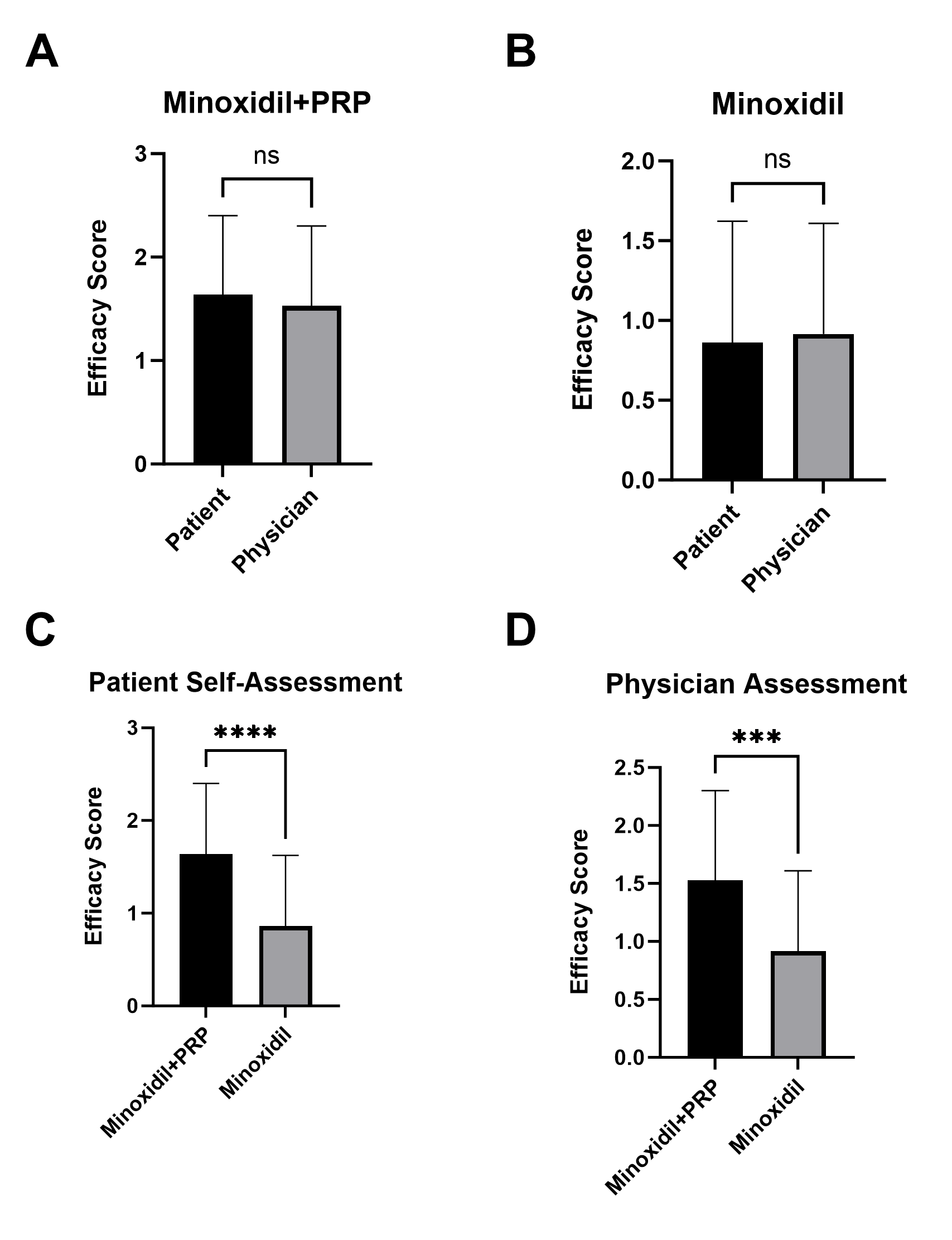

Supplement: Supplementary file 1 — Figure S1: Comparison of efficacy ratings between physicians and patients. (A) Physician vs. patient ratings in the minoxidil+PRP group. (B) Physician vs. patient ratings in the minoxidil‐only group. (C) Intergroup comparison of patient ratings. (D) Intergroup comparison of physician ratings. [file JOCD-25-e71078-s002.tiff]
