# Supplementary material for: Efficacy of Oxygen‐Enriched Platelet‐Rich Plasma Combined With Minoxidil in the Treatment of Androgenetic Alopecia: A Retrospective Study
Source: J Cosmet Dermatol. 2026 Jul 16;25(7):e71078. doi: 10.1111/jocd.71078 (PMC13377007; doi:10.1111/jocd.71078)
Supplement: Supplementary file 2 — Table S1: Baseline, final follow‐up visit, and change rates of quantitative dermoscopic parameters in the Minoxidil + PRP group and the Minoxidil group. Table S2: Adjusted generalized linear model analyses of change rates in quantitative dermoscopic parameters. [file JOCD-25-e71078-s001.docx]

**Supplement Table 1.** **Baseline, final follow-up visit, and change rates of quantitative dermoscopic parameters in the Minoxidil + PRP group and the Minoxidil group.**

| **Parameter** | **Minoxidil + PRP** | **Minoxidil** | ***P-value* ^a^** |
| --- | --- | --- | --- |
| Hair density (/0.922cm^2^) |  |  |  |
| Baseline | 176.45±43.18 | 188.57±31.50 | 0.100 |
| Final follow-up visit | 209.82±51.44 | 203.33±25.76 | 0.913 |
| Change rate^b^ | 1.19±0.08 | 1.09±0.13 | ＜0.001 |
| Hair diameter  (μm) |  |  |  |
| Baseline | 44.36±3.77 | 43.05±5.87 | 0.238 |
| Final follow-up visit | 51.64±4.27 | 47.74±6.73 | ＜0.001 |
| Change rate | 1.24±0.10 | 1.12±0.17 | 0.003 |
| Vellus hair ratio (%) |  |  |  |
| Baseline | 35.36±9.21 | 38.71±18.64 | 0.525 |
| Final follow-up visit | 26.36±6.73 | 30.23±16.62 | 0.444 |
| Change rate | 0.76±0.13 | 0.81±0.22 | 0.468 |
| Follicular density  (unit/0.922cm^2^) |  |  |  |
| Baseline | 88.67±11.57 | 84.14±8.41 | 0.074 |
| Final follow-up visit | 95.33±13.08 | 87.90±8.25 | ＜0.001 |
| Change rate | 1.13±0.18 | 1.05±0.11 | 0.012 |
| Mean number of hairs per follicular unit |  |  |  |
| Baseline | 1.81±0.16 | 1.78±0.14 | 0.475 |
| Final follow-up visit | 1.87±0.16 | 1.86±0.16 | 0.558 |
| Change rate | 1.05±0.05 | 1.05±0.08 | 0.808 |

a *P* values represent between-group comparisons at each time point and for change rates.

b Change rate was calculated individually for each participant and then summarized as mean±SD.

**Supplement Table 2.** **Adjusted generalized linear model analyses of change rates in quantitative dermoscopic parameters.**

| **Characteristic** | **Beta** | **95% CI** | ***P*-value** |
| --- | --- | --- | --- |
| **Hair Density** |  |  |  |
| Group |  |  |  |
| Minoxidil | Reference |  |  |
| Minoxidil+PRP | 0.067 | 0.012, 0.123 | 0.019 |
| Age | 0.001 | -0.002, 0.004 | 0.520 |
| Sex |  |  |  |
| Female | Reference |  |  |
| Male | -0.052 | -0.124, 0.020 | 0.160 |
| Duration | 0.001 | 0.000, 0.002 | 0.246 |
| Severity^a^ |  |  |  |
| Mild | Reference |  |  |
| Moderate | 0.046 | -0.026, 0.119 | 0.215 |
| Severe | -0.003 | -0.121, 0.115 | 0.957 |
| **Hair Diameter** |  |  |  |
| Group |  |  |  |
| Minoxidil | Reference |  |  |
| Minoxidil+PRP | 0.083 | 0.011, 0.156 | 0.028 |
| Age | 0.002 | -0.002, 0.006 | 0.331 |
| Sex |  |  |  |
| Female | Reference |  |  |
| Male | 0.024 | -0.071, 0.118 | 0.623 |
| Duration | 0.000 | -0.001, 0.002 | 0.728 |
| Severity |  |  |  |
| Mild | Reference |  |  |
| Moderate | -0.026 | -0.122, 0.069 | 0.591 |
| Severe | -0.028 | -0.184, 0.127 | 0.723 |
| **Follicular Density** |  |  |  |
| Group |  |  |  |
| Minoxidil | Reference |  |  |
| Minoxidil+PRP | 0.147 | 0.079, 0.215 | <0.001 |
| Age | -0.002 | -0.006, 0.001 | 0.218 |
| Sex |  |  |  |
| Female | Reference |  |  |
| Male | 0.068 | -0.020, 0.157 | 0.136 |
| Duration | 0.001 | -0.001, 0.002 | 0.426 |
| Severity |  |  |  |
| Mild | Reference |  |  |
| Moderate | -0.043 | -0.132, 0.047 | 0.353 |
| Severe | -0.004 | -0.150, 0.141 | 0.952 |
| **Vellus hair ratio** |  |  |  |
| Group |  |  |  |
| Minoxidil | Reference |  |  |
| Minoxidil+PRP | -0.044 | -0.128, 0.041 | 0.316 |
| Age | -0.001 | -0.006, 0.003 | 0.569 |
| Sex |  |  |  |
| Female | Reference |  |  |
| Male | 0.062 | -0.048, 0.172 | 0.271 |
| Duration | -0.001 | -0.003, 0.001 | 0.422 |
| \| Severity \| \| --- \| |  |  |  |
| Mild | Reference |  |  |
| Moderate | 0.011 | -0.100, 0.123 | 0.843 |
| Severe | -0.234 | -0.415, -0.054 | 0.013 |
| **Mean number of hairs per follicular unit** |  |  |  |
| Group |  |  |  |
| Minoxidil | Reference |  |  |
| Minoxidil+PRP | -0.007 | -0.041, 0.026 | 0.665 |
| Age | 0.000 | -0.002, 0.002 | 0.834 |
| Sex |  |  |  |
| Female | Reference |  |  |
| Male | -0.009 | -0.052, 0.035 | 0.695 |
| Duration | 0.000 | -0.001, 0.001 | 0.939 |
| \| Severity \| \| --- \| |  |  |  |
| Mild | Reference |  |  |
| Moderate | 0.016 | -0.028, 0.060 | 0.482 |
| Severe | -0.009 | -0.080, 0.062 | 0.803 |

^a^AGA severity was recoded as mild, moderate, or severe. In men, Norwood–Hamilton stages II–III were classified as mild, stage III vertex and stage IV as moderate, and stage V as severe. In women, Ludwig stages I, II, and III were classified as mild, moderate, and severe, respectively.

Data are presented as β coefficients with 95% confidence intervals and P values. The minoxidil monotherapy group and female sex were used as reference categories. Models were adjusted for treatment group, age, sex, and disease duration.
